# Deep learning identifies morphological determinants of sex differences in the pre-adolescent brain

**DOI:** 10.1016/j.neuroimage.2020.117293

**Published:** 2020-08-22

**Authors:** Ehsan Adeli, Qingyu Zhao, Natalie M. Zahr, Aimee Goldstone, Adolf Pfefferbaum, Edith V. Sullivan, Kilian M. Pohl

**Affiliations:** a Department of Psychiatry & Behavioral Sciences, Stanford University, Stanford, CA 94305, USA; b Center for Biomedical Sciences, SRI International, Menlo Park, CA 94025, USA

**Keywords:** Deep learning, Sex differences, Adolescents, Study confounders, Pubertal development, Cerebellum

## Abstract

The application of data-driven deep learning to identify sex differences in developing brain structures of pre-adolescents has heretofore not been accomplished. Here, the approach identifies sex differences by analyzing the minimally processed MRIs of the first 8144 participants (age 9 and 10 years) recruited by the Adolescent Brain Cognitive Development (ABCD) study. The identified pattern accounted for confounding factors (i.e., head size, age, puberty development, socioeconomic status) and comprised cerebellar (corpus medullare, lobules III, IV/V, and VI) and subcortical (pallidum, amygdala, hippocampus, parahippocampus, insula, putamen) structures. While these have been individually linked to expressing sex differences, a novel discovery was that their grouping accurately predicted the sex in individual pre-adolescents. Another novelty was relating differences specific to the cerebellum to pubertal development. Finally, we found that reducing the pattern to a single score not only accurately predicted sex but also correlated with cognitive behavior linked to working memory. The predictive power of this score and the constellation of identified brain structures provide evidence for sex differences in pre-adolescent neurodevelopment and may augment understanding of sex-specific vulnerability or resilience to psychiatric disorders and presage sex-linked learning disabilities.

## 1. Introduction

The concept of sex differences is based on biology and genetics. Since the 1930s (e.g., Pfeiffer, 1936), identifying sex differences in the Central Nervous System (CNS) has been explored in animal models (Becker and Koob, 2016; Galea et al., 2006; Goldstein et al., 2001; McEwen, 1983; Woodson and Gorski, 2000) and histology of postmortem human brain samples (Vogeley et al., 2000; Witelson et al., 2005; Witelson, 1989). More recently, in vivo neuroimaging (Fan et al., 2010; Filipek et al., 1994; Flaum et al., 1995; Goldstein et al., 2001; Hänggi et al., 2010; Sacher et al., 2013; Wang et al., 2012) and computer-based learning tools (Breslau et al., 2017; Feis et al., 2013; Nieuwenhuis et al., 2017; Van Putten et al., 2018; Xin et al., 2019) have been implemented in the search for a CNS basis of sexual differentiation. Beyond sex-linked risks for disease (Brie et al., 2019; Egloff et al., 2018; Jahanshad and Thompson, 2017; Lind et al., 2017; Retico et al., 2016; Vogeley et al., 2000), this search is motivated by adolescence being a period of particular vulnerability to the emergence of sex-linked neuropsychiatric disorders such as schizophrenia (Vogeley et al., 2000; Womer et al., 2016) and autism (Golarai et al., 2006; Liu et al., 2016; Pierce et al., 2019; Retico et al., 2016; Strickler et al., 2020), which have a higher prevalence in boys than girls, and depression, which girls by age 15 develop twice as likely as boys (Breslau et al., 2017; Cyranowski et al., 2000).

In vivo structural magnetic resonance imaging (MRI) studies characterize brain development as following heterogeneous growth trajectories (Giedd, 2004; Petrican et al., 2017) during which sex-specific behaviors emerge (Johnson and Meade, 1987). While physical signs of sex differences are present at birth (Gilmore et al., 2007), brain structural and functional differences between the sexes continue to develop over childhood through late adolescence (Giedd et al., 2015; Mankiw et al., 2017; Pfefferbaum et al., 2016, 2018; Tamnes et al., 2017). For example, both cortical and subcortical gray matter volumes exhibit inverted U-shaped trajectories reflecting growth followed by synaptic pruning, with boys showing a slightly larger rate of change throughout childhood and adolescence than girls (Lenroot et al., 2007b). With respect to white matter, the volume increases with age in both sexes, but boys generally show a more rapid increase during adolescence (Lenroot et al., 2007b). These sex specific changes in brain structure during adolescence (Wierenga et al., 2018a) are accompanied with asexual developments, such as structural volume (Aubert-Broche et al., 2013; Ducharme et al., 2015; Herting et al., 2018; Mills et al., 2012; Narvacan et al., 2017; Vijayakumar et al., 2016), cortical thickness (Vijayakumar et al., 2016), cortical surface area (Ducharme et al., 2015; Vijayakumar et al., 2016), individual’s behavior (Wierenga et al., 2014), and testosterone effects (Wierenga et al., 2018a).

Many of the differences in brain development between the sexes are actually linked to head size (Ruigrok et al., 2014; Sanchis Segura et al., 2018). As boys on average have larger brains than girls, identifying sex differences in the brain beyond head size is challenging and might explain the inconsistent findings in the literature. For example, whether sex differences are present within the corpus callosum has been a matter of debate (Etchell et al., 2018; Jahanshad and Thompson, 2017; Luders et al., 2014; Sawyer et al., 2018; Sullivan et al., 2001). Beyond properly accounting for head size (Luders et al., 2014; Perlaki et al., 2014; Pfefferbaum et al., 2016; Sanchis Segura et al., 2018), discrepancies in findings may be due to small sample sizes (Button et al., 2013; Filipek et al., 1994; Lenroot et al., 2007a), wide age distributions (sometimes across several decades so age-specific sex differences are obscured) (Etchell et al., 2018; Kim et al., 2012), or a priori assumptions that reduce the rich information encoded in MRIs to a few brain measurements (e.g., volumes of a limited number of brain regions of interest (ROIs)) (Etchell et al., 2018; Xin et al., 2019). The study presented herein accounts for these issues by building on recent advancements in the field of deep learning (Esmaeilzadeh et al., 2018; Krizhevsky et al., 2012; Van Putten et al., 2018) to identify patterns not driven by study confounders, which are extraneous variables (such as age) that may induce undesired class differences if not properly controlled.

**Fig. 1.**
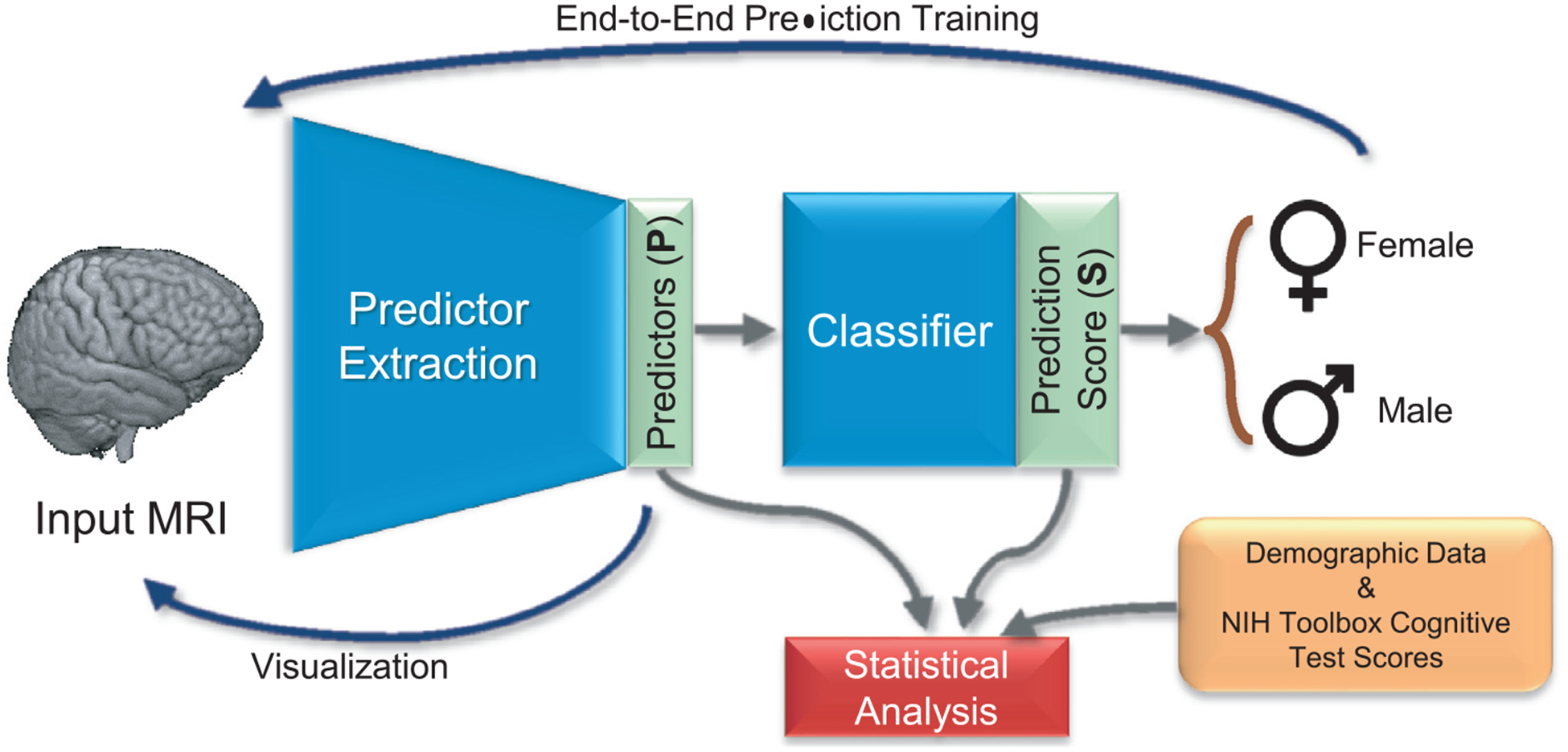
Overview of the proposed analysis. The convolutional neural network (CNN) automatically extracts predictors (**P**) from the minimally processed MRI. Based on **P**, the classifier computes a prediction score (**S**) that assigns the MRI to either sex. This deep learning analysis operates directly on voxel-level data omitting any hypothesis or assumption related to brain regions or tissue measurements (like regional volumes). Statistical analysis relates obtained results to NIH Toolbox cognitive test scores, creates confounder-free visualization of the patterns predicting sex (a.k.a. saliency map), and examines volume scores of those regions that contribute significantly to the prediction according to the saliency map.

Specifically, we present a deep learning framework (see Fig. 1) predicting sex from the minimally processed T1-weighted (T1w) MRIs (Hagler et al., 2019) of 8144 pre-adolescents (ages 9 and 10 years) of the ABCD study (http://abcdstudy.org). The variance in the prediction scores is related to the cognition test scores of the National Institutes of Health (NIH) Toolbox^®^ (Luciana et al., 2018). Finally, we qualitatively assess the average *saliency map* (Simonyan et al., 2014) across all MRIs, which encodes the contribution of each voxel of the MRI in predicting sex while removing the effects driven by the confounders, i.e., age and pubertal and socioeconomic status.

## 2. Materials and methods

### 2.1. ABCD participants and study design

The model was evaluated on data collected by the ABCD study (http://abcdstudy.org). Demographic information (Table 1), cognitive test scores from the NIH toolbox (Table 2, details are explained in Appendix A), and T1-weighted (T1w) MR images (Hagler et al., 2019) from 8670 participants were distributed by the ABCD-Neurocognitive Prediction Challenge (ABCD-NP-Challenge 2019) (Pohl et al., 2019) via the National Database for Autism Research (NDAR) portal (Release 2.0), of which 8144 subjects contained the data needed for this analysis. Socioeconomic status (SES) was estimated by identifying the maximum level of education across parents/guardians as done elsewhere (Sullivan et al., 2016). Pubertal status was determined by self-assessment with the Pubertal Development Scale (PDS) (Carskadon and Acebo, 1993; Petersen et al., 1988), a validated measure of pubertal stage that shows modest concordance with a physical exam and that correlates with basal gonadal hormone levels. An average PDS was calculated for each participant by adding up scales on five self-reports obtained from parents’ responses to a questionnaire, where each scale ranged from 1 to 4. Based on this computation, PDS categorized ABCD youth as either (1) pre-pubertal, (2) early-pubertal, (3) mid-pubertal, (4) late-pubertal (5) post-pubertal. Participants of multiple ethnicities were categorized according to their minority ethnicity (e.g., a report of Asian and Caucasian was classified as Asian) (Pfefferbaum et al., 2016). Body Mass Index (BMI) was calculated based on published methods (Freedman et al., 2017). Observed Sex for all the participants was defined as the sex at birth.

Recruitment for the ABCD study closely represented the general U.S. population of 9 and 10 year-old children with respect to key demographic variables including sex, ethnicity, household income, parental education, and parental marital status (Thompson et al., 2019). Parents provided informed consent and were fluent in either English or Spanish; children had to be fluent in English and provide assent for participation. Exclusionary criteria included poor English-language proficiency; the presence of severe sensory, intellectual, medical, or neurological issues that would affect the validity of data or ability to comply with the protocol; and contraindications to MRI (see Garavan et al., 2018) for complete description of details regarding recruitment and inclusion/exclusion criteria).

### 2.2. MRI data acquisition and processing

Details on T1w-MRI acquisition are provided by https://abcdstudy.org/images/Protocol_Imaging_Sequences.pdf Processing of T1w-MRI were subjected to the ABCD minimal-processing pipeline (Hagler et al., 2019) followed by noise removal (Coupe et al., 2008) and field-inhomogeneity correction via N4ITK (Version 2.1.0) (Tustison et al., 2010). Brain masks were determined via majority voting (Rohlfing et al., 2004) over the segmentations generated by applying the following tools to both bias and non-bias corrected T1w-images: FSL BET (Version 5.0.6) (Smith, 2002), AFNI 3dSkullStrip (Version AFNI_2011_12_21_1014) (Cox, 1996), FreeSurfer mri-gcut (Version 5.3.0) (Sadananthan et al., 2010), and Robust Brain Extraction (ROBEX) (Version 1.2) (Iglesias et al., 2011). The resulting brain mask was used to refine correction for image-inhomogeneity and skull stripping. MRIs were then affinely registered to the SRI24 template (Rohlfing et al., 2010), down-sampled to 2 mm isotropic voxel size, and re-scaled to 64 × 64 × 64 volumes. The affine registrations ensured that all MRIs of the ABCD study had similar head size as measured by supratentorium volume (svol) (see also Table 1 for the resulting insignificant difference in head size between boys and girls).

Fig. 1 outlines the deep learning framework used to predict sex from minimally processed MRI data. The framework was composed of a Predictor/Extractor and a Classifier (Esmaeilzadeh et al., 2018; Nie et al., 2016). The Predictor/Extractor identified a set of Predictor variables **P** = {**P**^1^, **P**^2^, …, **P**^*M*^} from MR images based on a deep convolutional network (Krizhevsky et al., 2012). The Classifier was a set of fully connected layers reducing **P** into a continuous Prediction Score **S**, which was the probability *π* computed by the classifier of an MRI being associated with a girl (i.e., *π*(girl) = **S**) or a boy (i.e., *π*(boy) = 1 − **S**). Appendix B provides a more in-depth description of the deep learning architecture.

The prediction accuracy of the model was determined in two steps. Assuming that sex affects the brain bilateral (Hill et al., 2014; Hirnstein et al., 2019; Phinyomark et al., 2014; Román et al., 1989; Weinhandl et al., 2010) and to simplify the interpretation of the findings, the left hemisphere was first flipped to create a 2nd “right ” hemisphere. Then, 5-fold cross-validation (Kohavi, 1995) was performed by splitting the data based on subjects. At each iteration of the cross-validation, the four folds of the data used for training were first augmented to ensure that the learning was based on a balanced and sufficient number of boys and girls (Oksuz et al., 2019), *i.e*, 5000 for each group. Data augmentation consisted of applying random rigid transformations (within one voxel shifting and 1° rotation along the three axes) to the minimally processed (and flipped) MRIs. On this augmented data set, the entire deep model, which included the predictor extractor and the classifier, was trained from scratch in an end-to-end manner (Esmaeilzadeh et al., 2018). Next, the prediction of the individual’s sex was recorded on the fifth fold (which was not augmented) by computing the average prediction score (**S**) across both hemispheres. The training and testing processes were repeated until the prediction score was reported for each subject. The average accuracy of the method on all folds was then computed by first binarizing **S** of each participant to 1 (girl) or 0 (boy) and then comparing the predictions to their observed sex via commonly used metrics: balanced classification accuracy (Park et al., 2018) (a.k.a. accuracy), true positive rate, false positive rate, and the area under the receiving operating characteristic curve.

To put the prediction accuracy in perspective and compare with widely used machine learning methods, the cross-validation was repeated with respect to logistic regression (Adeli et al., 2020; Kleinbaum and Klein, 2002), support vector machines (Chang and Lin, 2011) and random forest (Liaw and Wiener, 2002) applied to the volumes of 116 brain ROIs defined according to the SRI24 atlas (Rohlfing et al., 2010). Measuring the volumes of ROIs consisted of non-rigidly registering the SRI24 atlas to each brain-size corrected MRI via ANTS (Version: 2.1.0) (Avants et al., 2008) and overlaying parcellations with the tissue segmentations from Atropos (Avants et al., 2011). The experiment was repeated using the 906 regional scores generated by Freesurfer based on the Destrieux atlas (Destrieux et al., 2010), which were provided by the ABCD Study Release 2.0 (http://abcdstudy.org). These regional scores consisted of cortical thickness, sulcal depth, surface area, and volume of cortical ROIs and the average T1 intensities within the white and gray matter.

**Fig. 2.**
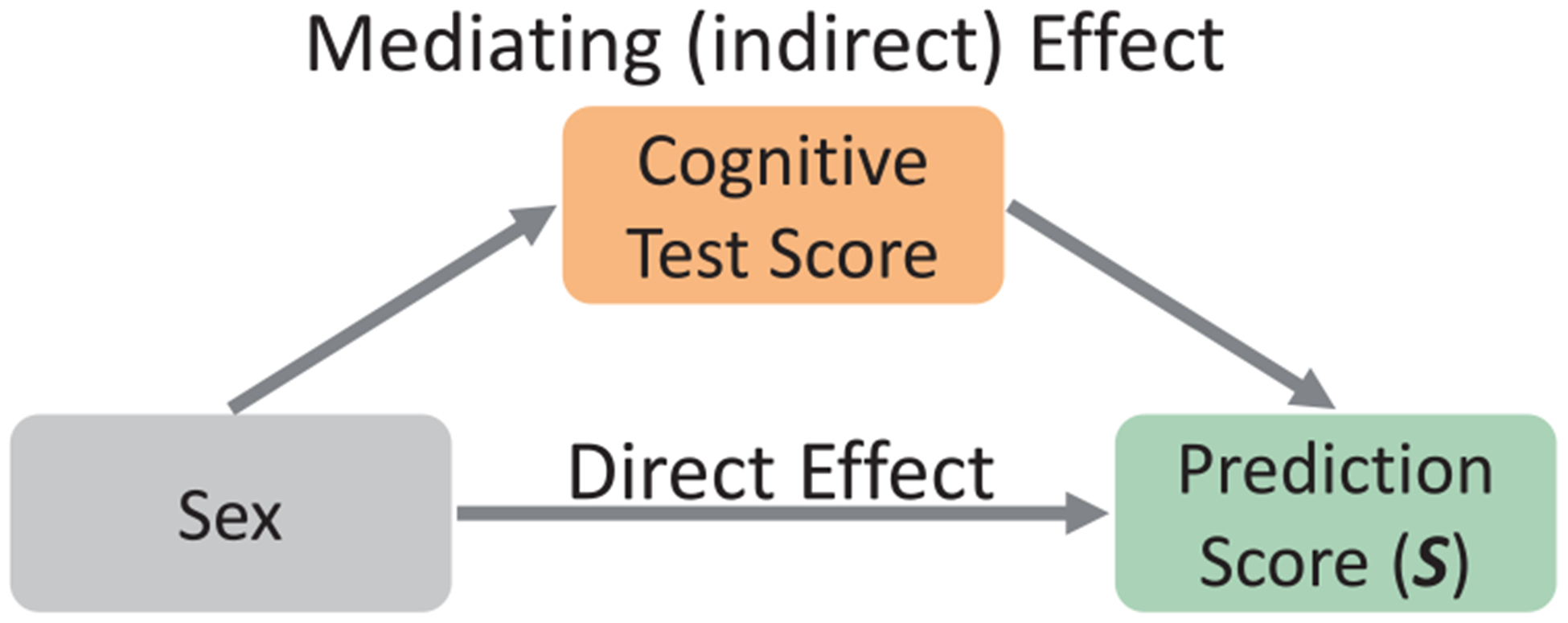
Mediation analysis to observe how much of the variance in the prediction score was explained by the observed sex and how much was influenced by the NIH toolbox score.

In addition to the comparison to other methods, a sex-agnostic test correlated the prediction score **S** of the individuals with the test scores of the age-corrected NIH toolbox (significant *p*-value *<* 0.05 according to Pearson’s *R*). Identifying variance in the prediction score partially induced by an NIH toolbox score (Fig. 2) was done via the partial mediation model (Baron and Kenny, 1986). Partial mediation required that (1) observed sex significantly correlated with the NIH toolbox score; (2) the NIH toolbox score significantly correlated with the prediction score when accounting for observed sex as an additional covariate; and (3) the correlation between observed sex and the prediction score was significantly reduced (*p*-value inferred from a permutation test of 10,000 permutations) when accounting for the NIH toolbox score as an additional covariate.

Finally, we performed bootstrapping (5 runs) to determine the effects of PDS (the most significant confounder of this study according to Table 1) on the sex predictions of our approach. Each of the 5 runs was defined by 5-fold cross-validation consisting of a unique random split of the data into 5-folds. The correctly classified subjects in all 5 runs were assigned to one group, and the ones that were incorrectly classified in all 5 runs were assigned to a second group. For each sex separately, differences in the PDS between the two groups were defined by the *p*-value of the *χ*^2^ test (Pearson, 1900). Across the two groups, the prediction accuracy (for both boys and girls) was determined for cohorts confined to the same PDS. We then reported on the cohorts with a sufficient number of samples for each sex, which were the cohorts for PDS 1, 2, and 3.

All methods were implemented using Python 3.7.0 and its libraries including SciPy 1.1.0, NumPy 1.15.1, Scikit-Learn 0.19.2, pygrowup 0.8.2 toolbox (pygrowup, 2017), Tensorflow 1.7.0 (Abadi et al., 2016), and Keras 2.2.2 (Gulli and Pal, 2017). The codes of our deep learning implementation are publicly available at https://github.com/QingyuZhao/Confounder-Aware-CNN-Visualization and the tests at https://github.com/eadeli/ABCD_SexDiff.

### 2.3. Identifying confounder-free patterns and ROIs relevant to sex

To derive a single pattern informative for identifying sex differences, we re-trained our proposed approach on the entire dataset. For each participant, the discriminative power of each voxel to predict sex was recorded using a saliency map (Simonyan et al., 2014). The initial salience map was computed by applying the minimally processed and flipped MRI to the trained prediction model and then performing back-propagation (Kotikalapudi and contributors, 2017). Note, saliency computation did not require data augmentation nor estimating prediction accuracy.

Next, the map was further corrected for the effects of potential confounders on the decision process of the model. Confounders were demographic factors significantly different between sexes according to Table 1, i.e., age (**z**^*age*^), PDS (**z**^*pds*^), and SES (**z**^*ses*^). To determine if a confounder significantly influenced the decision process of **S**, a general linear model (GLM) (Madsen and Thyregod, 2010) was fit across all samples with respect to each predictor variable **P**^*j*^ of **P** :

(1) $$P^{j}=\beta_{0}+\beta_{1}S+\beta_{2}z^{\text{pds}}+\beta_{3}z^{\text{age}}+\beta_{4}z^{\text{ses}}.$$

If the predictor variable **P**^*j*^ of the GLM significantly correlated (*p* ≤ 0.05) with one of the demographic variables, the predictor was considered confounded and omitted from computing the saliency maps. The lenient *p*-value threshold of 0.05 was not corrected for multiple comparison as we wanted our analysis to be sensitive towards identifying confounded predictors so that the resulting pattern accurately represented sex differences. The pattern encoding the relevance of each voxel in predicting sex was defined by the average across the confounder-free saliency maps of all participants. Conversely, a pattern encoding the effect of a specific confounder was created by computing the saliency maps based on confounded predictors.

To relate the identified voxels to previously defined brain ROIs (using SRI24 atlas, Rohlfing et al., 2010), we computed the average saliency value of each ROI from the confounder-free saliency map of each participant. For each ROI, follow-up *t*-tests evaluated whether the average saliency value within that region was significantly different between groups (*p*-value *<* 0.05 with Bonferroni multiple comparison correction Shaffer, 1995).

## 3. Results

The accuracy of the prediction score in correctly assigning MRIs to either sex was 89.6% (Receiver operating characteristic curve in Appendix C), which was significantly better than chance (*p <* 0.001 according to a Fisher exact test, Fisher, 1935). The prediction accuracy was stable across 5 runs of 5-fold cross-validation based on random splitting of folds (89.6% ± 0.13%) but was slightly lower (87.3%) on a subset of 2464 boys and 2464 girls matched on head size (matched according to Adeli et al., 2018). Furthermore, the True Positive Rate (TPR) of the deep learning model was 87.4% and True Negative Rate (TNR) was 91.5% (girls = 1, boys = 0). Compared with the correctly classified pre-adolescents, misclassified boys had significantly higher PDS while misclassified girls had significantly lower PDS (*p*-value *<* 10^−6^ according to *χ*^2^ test). The prediction confined to individuals with the same PDS was 88.9% for participants with PDS = 1, 89.5% for PDS = 2, and 90.1% for PDS = 3.

**Fig. 3.**
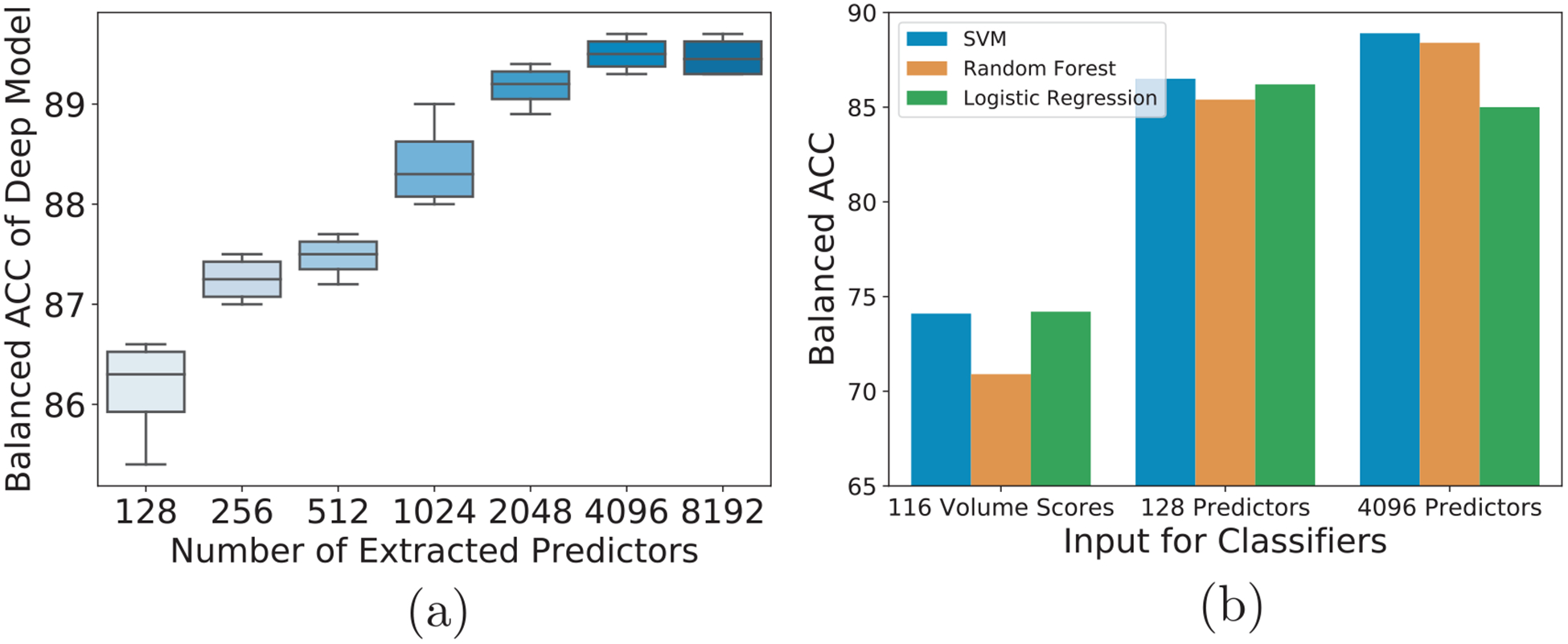
Results of the deep learning model predicting sex with different numbers of predictors (a), and different classifiers (b).

The prediction of our approach was significantly more accurate (Delong test, DeLong et al., 1988, *p*-value *<* 0.001) than the results reported by Logistic Regression, Support Vector Machine, and Random Forest applied to the 116 ROI volume measures or the 906 Destrieux parcellation measures (see Table 3). To gain a better understanding of this improvement, we recomputed the accuracy of our model across 5 runs of 5-fold cross-validation with respect to the number of predictors. The average accuracy remained relatively high (86.5%) even when extracting only 128 predictors from each MRI (see Fig. 3 (a)). Furthermore, similarly high accuracy was achieved by the other approaches when trained on the predictors extracted by our deep model (Fig. 3 (b)).

**Fig. 4.**
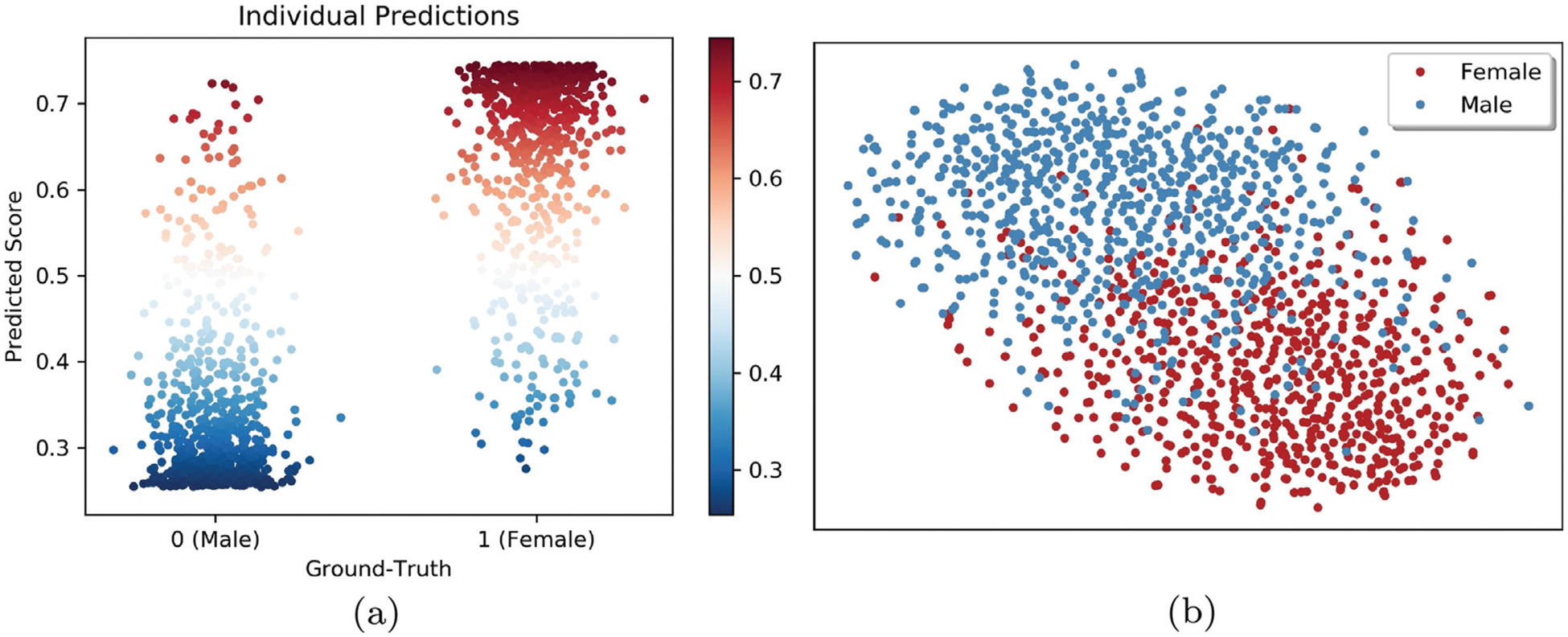
Visualization of **Predictors** and the **Prediction Score** as determined by the deep learning model. (a) Prediction Score (**S**) of each participant as a function of their observed sex. These two figures show that our deep learning model can effectively reduce the MRIs to a vector of predictors (**P**) and then to a scalar value (**S**) that distinguishes girls from boys. (b) *t*-Distributed Stochastic Neighbor Embedding (tSNE) (Maaten and Hinton, 2008) projection of extracted Predictors (**P**) in 2D space. Each point indicates one adolescent; color represents sex. The axes show the relative location of each individual with respect to their neighbors in 2D with neighborhoods reflecting those of the high dimensional space (according to Maaten and Hinton, 2008).

A visual confirmation of the significant prediction accuracy of our model were the two distinct distributions shown in Fig. 4 (a), which plotted the Prediction Score (**S**) of each participant as a function of their observed sex. Furthermore, projecting the high dimensional Predictors (**P**) learned from one training run into 2D via the *t*-distributed Stochastic Neighbor Embedding (tSNE) (Maaten and Hinton, 2008) also resulted in a cluster for boys and a separate one for girls (Fig. 4 (b)).

**Fig. 5.**
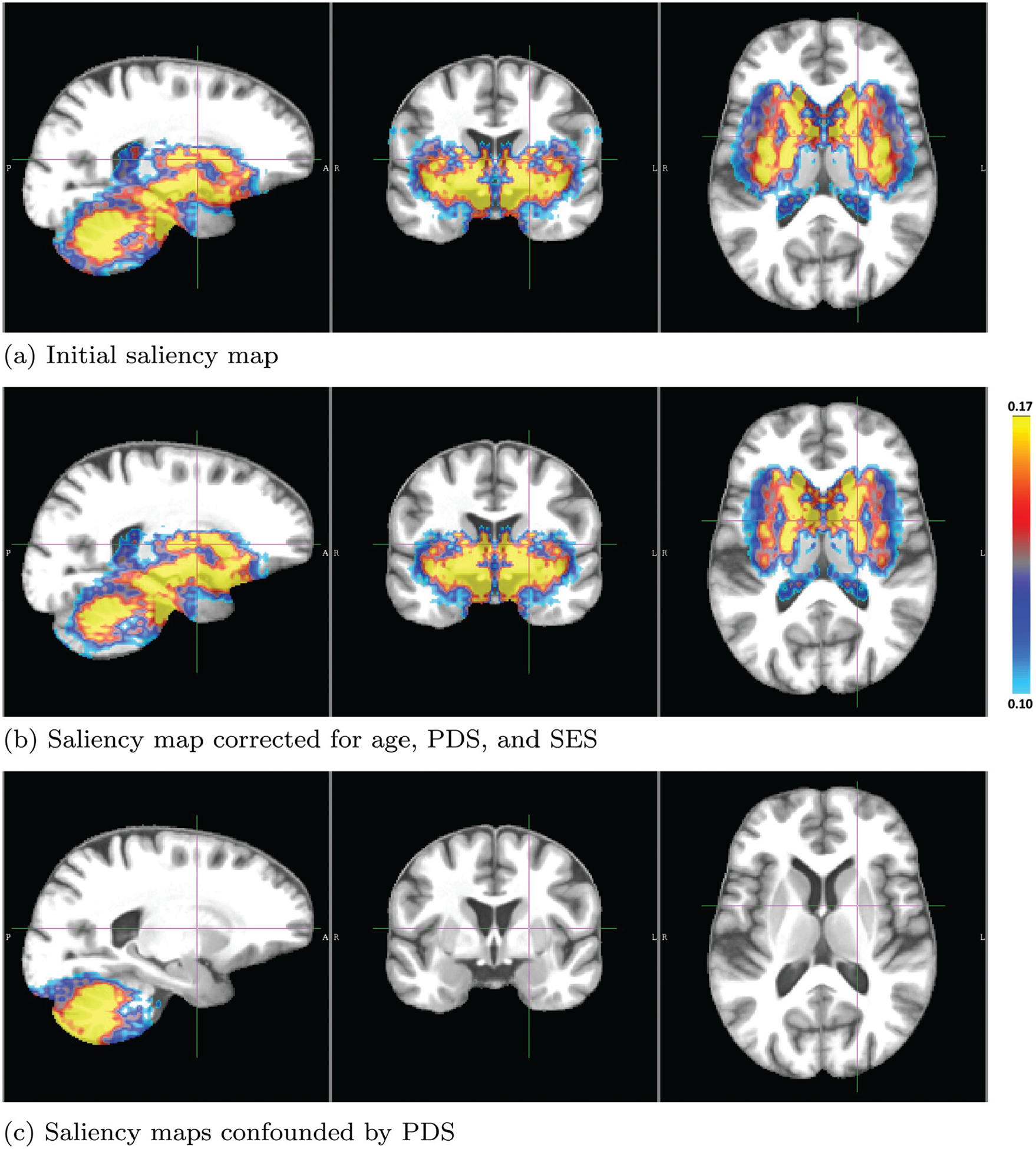
Saliency maps defining predictive brain areas for distinguishing boys from girls in the ABCD study; (a) original and (b) corrected for confounding factors. In the developing brain of 9 and 10-year-olds, the factors distinguishing boys from girls mainly lie in the subcortical and cerebellar regions. (c) Regional brain pattern of sex differences confounded by PDS. Note, computing saliency maps requires scaling of the maps so that the resulting importance values are only meaningful within one saliency map but cannot be directly compared across maps.

**Fig. 6.**
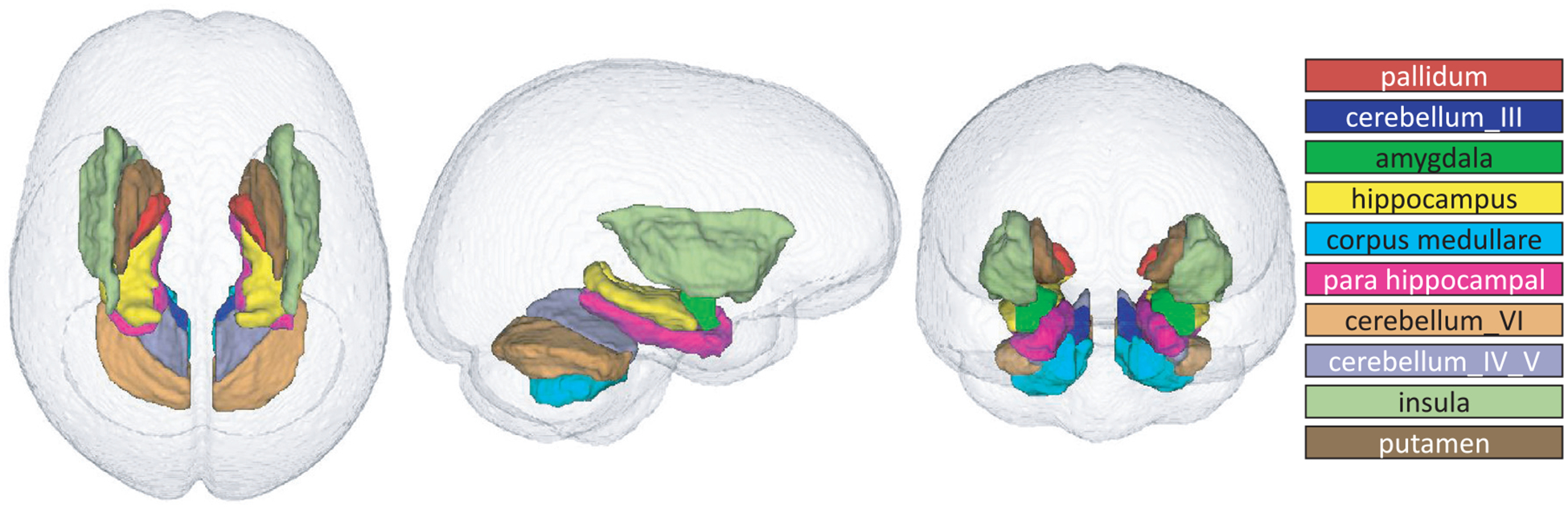
Top 10 regions relevant for distinguishing sex as determined by the deep learning framework. Some of these regions are smaller in girls (cerebellar lobules III and IV/V, amygdala; and insula, pallidum, para hippocampus, and putamen), while hippocampus, corpus medullare, and cerebellar lobule VI are smaller in boys. *p*-Values of group differences of ROI volumes were calculated using two sample *t*-test. NS denotes *not significant*

Fig. 5 (a) visualizes the initial saliency map with voxel values above 0.1 before correcting for confounders. The highlighted area significantly contributed to predicting sex, which partly consisted of the temporal lobes, subcortical regions, cerebellum, and corresponding white matter. Fig. 5 (b) shows the area of sexual differentiation according to the confounder-free saliency map (i.e., with age, PDS, and SES removed), which is more spatially concentrated than the initial saliency map (Fig. 5 (a)). According to the confounder-free saliency values, the 10 ROIs most relevant for predicting sex were insula, pallidum, para hippocampus, and putamen (larger in boys than girls); hippocampus, corpus medullare, and cerebellum VI (larger in girls than boys) (Fig. 6). Although deep learning identified insula, amygdala, and cerebellar lobules III and IV/V as significant predictors of sex, their volume differences by sex were not forthcoming. The cerebellum was also the region mostly confounded by PDS (Fig. 5 (c)), the most significant confounder in the model.

Table 4 lists the correlation and mediation effect of NIH toolbox scores with respect to the prediction score **S** Significant correlations (*p*-value *<* 0.05) between **S** and NIH toolbox scores were confined to the List Sorting Working Memory Test, Pattern Comparison Processing Speed, Picture Sequence Memory Test, and Picture Vocabulary Test. Further, a partial mediation model examined whether the NIH toolbox scores could partially explain the variance in **S** in addition to the observed sex (Fig. 2). Only the List Sorting Working Memory Test score met the 3 significance conditions of the mediation model (*p*-value *<* 0.05): (1) observed sex significantly correlated with the NIH toolbox score; (2) the NIH toolbox score significantly correlated with **S** when accounting for observed sex as an additional covariate; and (3) the correlation between observed sex and **S** was significantly reduced when accounting for the NIH toolbox score as an additional covariate.

## 4. Discussion

The deep learning model presented herein not only successfully predicted the sex of 8144 pre-adolescents from (head-size normalized) T1w MRI but also was more accurate than several other commonly used machine learning approaches, e.g., logistic regression, support vector machine, and random forest. While these machine learning approaches relied on *a priori* defined regional measurements (as is commonly used for neuroscience studies, Adeli et al., 2018; Aubert-Broche et al., 2013; Chung et al., 2005; Green et al., 2016; Kim et al., 2012), the improved accuracy of the deep learning model was mostly due to its ability to simultaneously extract predictors directly from the MRIs and perform classification (see Fig. 3). A novel discovery of that search for discriminative information was that sex could be accurately predicted in individual pre-adolescents through a pattern composed of subcortical and cerebellar regions. Also unknown for pre-adolescence was that the cerebellum was most strongly affected by PDS, the most significant confounder of the study. Finally, reducing the pattern to a single score revealed that its variance was not only explained by sex but also by cognitive behavior linked to working memory.

Critical for interpreting the pattern was the notion that sex differences on brain structure are bilateral (Hirnstein et al., 2019; Phinyomark et al., 2014; Román et al., 1989; Weinhandl et al., 2010). We modeled that by ‘flipping’ the left hemisphere and then training the algorithm on two ‘right’ hemispheres for each subject. When omitting flipping, the prediction accuracy was 89.1% when just trained on the left hemisphere, 88.5% when only trained on the right hemisphere, and 90.1% when trained on both hemispheres (omitting flipping). These accuracy scores were insignificantly different (*p >* 0.1; DeLong’s test) from those of the ‘flipped’ approach confirming the bilateral nature of sex differences.

Another critical aspect in analyzing the pattern was computing a saliency map that displayed brain areas exhibiting sex differences while accounting for confounders; something that had not been attempted by prior data-driven analyses (Feis et al., 2013; Nieuwenhuis et al., 2017; Ruigrok et al., 2014; Van Putten et al., 2018; Xin et al., 2019). Removing confounding effects after training a machine learning model is potentially a more conservative approach compared with removing effects through preprocessing (e.g., matching), i.e., before the training. Unlike removing confounding effects after training, preprocessing generally cannot completely remove those effects so that learning approaches can still leverage the remaining confounding effects to ‘improve’ predictions (Park et al., 2018). Of the three confounders considered, PDS was the most significant one, which was generally larger in girls than in boys within the pre-adolescent age range (Table 1). While misclassified boys had significantly higher PDS and misclassified girls had significantly lower PDS than correctly classified individuals of the same sex, the prediction accuracy of our deep learning model was not affected by PDS as the overall accuracy of 89.6% remained stable when confining the evaluation to individuals with the same PDS. The region most confounded by PDS was the cerebellum (Fig. 5 (c)) suggesting that pubertal status may be specifically associated with cerebellum development at this young age. This hypothesis is difficult to test on the baseline data of ABCD as the majority (~ 73%) of individuals were categorized as pre- or early pubescent. However, as the ABCD cohort ages, the variability in PDS will be considerably greater, and as such, will allow us to explore in more detail the potential interaction between sex and puberty in terms of cerebellar development.

In addition to the relationship to PDS, structures of the cerebellum were critical to predicting sex in individual, which is inline with a number of adult studies (Chung et al., 2005; Fan et al., 2010; Raz et al., 2001; Tiemeier et al., 2010). However, sex differences in cerebellar volume became generally negligible once studies corrected for intracranial volume (e.g., Nopoulos et al., 2000; Sullivan et al., 2020; Szabo et al., 2003). More specifically, the corpus medullare of the cerebellum in this study was significantly larger in girls than boys. By contrast, the longitudinal study by Tiemeier et al., 2010 did not detect significant sex differences in the corpus medullare but reported that total cerebellar volume was larger in boys than girls, and that this total volume peaked at age 15.6 years in boys and at age 11.8 years in girls. The discrepancy in age range of the participants between that study (spanning pre-adolescents to young adults) and our analysis (ages 9 and 10 years) might reflect variance in cerebellar developmental trajectories during critical developmental years. Indeed, a recent review of the literature on language and brain development concluded that sex differences were most often found in studies limited to tight age ranges (Etchell et al., 2018). Sex differences in regional brain volumes may be apparent in some but negligible in other developmental stages, likely due to different rates of brain maturation between girls and boys (Luna et al., 2004).

Of the predictive regions within the subcortex, the hippocampus was larger in girls than boys after correcting for head size (see Fig. 6). The hippocampus has often been associated with sex-specific differences in memory and learning in adolescence (Aggleton et al., 2010; Pilly et al., 2018). This observation comports with the finding that girls participating in the ABCD study had significantly higher scores on the NIH Toolbox Picture Sequence Memory Test, which is a validated measure of episodic memory (Dikmen et al., 2014a). The finding that girls had relatively larger hippocampi than boys is also supported by MRI studies of young adults (Filipek et al., 1994; Frodl et al., 2002; Szabo et al., 2003) that linked sex differences in hippocampal volume to hormonal responsivity (Giedd et al., 1996; Teicher et al., 2003) and memory performance (Hill et al., 2014; Trenerry et al., 1995; Young et al., 2013). Other studies noted relations between hippocampal volumes and clinical characteristics of psychiatric disorders (Egloff et al., 2018; Frodl et al., 2002; Yang et al., 2017), where sleep disturbances are more severe (Yang et al., 2017), depressive episodes are more frequent and longer, and higher frequency of migraines occurs in depressed female compared to depressed male patients (Saunders et al., 2014).

Other regions relevant for predicting sex included putamen, pallidum, and amygdala. These regions are frequently noted with reference to sex differences in brain maturation. An early imaging study of children aged 4–18 years suggested that while the caudate is relatively larger in girls, the pallidum is larger in boys (Giedd et al., 1997). A more recent study based on data from the Pediatric Imaging, Neurocognition, and Genetics (PING) study with 1234 participants (ages 3 to 21 years) (Wierenga et al., 2018b) showed that volumes of putamen and pallidum had greater variance in boys than girls: these differences may contribute to the variability in cognition and general intelligence in developing boys (Arden and Plomin, 2006; Baye and Monseur, 2016). Likewise, the amygdala has been linked to sex differences in animal and human studies across the lifespan (Blume et al., 2017; Green et al., 2016). A surface-based modeling approach showed that men had a larger mean radius of amygdala subregions than women (Kim et al., 2012). Further, sex differences in amygdala volume may contribute to the expression of selective psychotic disorders occurring more commonly in men than women (Blume et al., 2017) and depressive disorders, which are more common in women (Breslau et al., 2017; Cyranowski et al., 2000).

Like the amygadala, the insula was important for predicting sex but its volume was insignificantly different between the two cohorts. Functional studies have frequently shown the significant role of these two regions in working memory performance (Menon and Uddin, 2010). Interestingly in our study, sex prediction by the deep learning model was mediated by the List Sorting Working Memory test score, which was higher for boys than girls (see Table 4). These results suggest that the deep learning approach of directly analyzing intensity values at a voxel level is potentially more powerful in extracting morphological characteristics linked to cognitive differences between the sexes than traditional approaches that focus on specific measurements.

In addition to the mediation analysis, the predictive score was significantly correlated to most of the cognitive scores by the NIH Toolbox. These early and pervasive sex differences in neurocognitive measures echoed those identified on the 10,000 youth of the Philadelphia Neurodevelopmental Cohort (PNC) (Gur and Gur, 2016), in which girls performed better than boys on tasks assessing verbal memory and social cognition, whereas boys excelled on spatial processing and motor speed (Gur and Gur, 2017; 2016). Similar results were reported with the National Consortium on Alcohol and Neurodevelopment in Adolescence (NCANDA) data, whose cognitive test battery included those of the PNC study (Sullivan et al., 2016). Further consistency in sex differences on performance is forthcoming between our results and those published by the PING study, which, like the ABCD study, used the NIH Toolbox Battery. The PING study found that girls performed better than boys on tests assessing cognitive flexibility, problem solving, and episodic memory, whereas boys performed better on a list sorting task, assessing working memory for sorting and sequencing information (Akshoomoff et al., 2014). Taken together, the consistency of sex differences in the development of component processes of selective cognitive skills transcended cohort differences and specific testing materials, which provide evidence for generalization of these identified sex differences.

### Limitation.

Our analysis did not detect significant sex differences in the cortex possibly because the MRIs were affinely aligned to a template, thereby minimizing headsize differences. While a common practice in end-to-end training (Bäckström et al., 2018; Esmaeilzadeh et al., 2018), affine registration might poorly align the cortical gyri and sulci given their high inter-subject variability (Llera et al., 2019). Non-rigid registration achieves better voxel-wise correspondence across MRIs enabling learning algorithms to focus on fine-grained regional cues (Lin et al., 2018; Liu et al., 2018). Now identifying cues differentiating between groups highly depends on the ‘stiffness’ of the deformation field (Murphy et al., 2016; Wittek et al., 2010), which can substantially modify the shape and appearance of brain structures. One possible data driven approach for setting the stiffness with respect to the cortex is to first parcellate the structure (via a surface based segmentation tool, Dale et al., 1999; Onofrey et al., 2018; Zhao et al., 2019) and then perform an ROI-based registration for the whole brain (such as Lopez-Garcia et al., 2006; Yi et al., 2006). As any of these registration can negatively affect analysis, their effect on our deep learning findings needs to be further investigated.

## 5. Conclusion

The voxel-level analysis on the large number (*N* = 8144) of pre-adolescents (age 9 and 10) confirmed and extended the common finding of smaller neuroimaging studies that cerebellum and subcortical structures (including hippocampus, amygdala, pallidum, and putamen) differed in size between boys and girls. Not known before, however, was that the constellation of those brain structures accurately predicted the sex of individual pre-adolescents. The predictive power of the pattern provides evidence for sex differences in pre-adolescent, pubertal development, which may show even greater differentiation as the cohort ages. Tracking these disparities is a normative process that could augment understanding of sex-specific vulnerability or resilience to psychiatric disorders and presage sex-linked learning disabilities.

## Figures and Tables

**Table 1:** Demographic information (mean ± standard deviation).

Measure	Female (F)	Male (M)	*p*-value^a^	Group difference
Total subjects	3895	4249	-	-
Age (years)	9.92 ± 0.62	9.95 ± 0.62	0.04	F < M
Head size^b^ (svol cm^3^)	1341.5 ± 15.3	1342.0 ± 16.0	NS	F=M
Socioeconomic Status (SES)	18.0 ± 66.8	18.5 ± 67.8	NS	F = M
Pubertal Development Scale (PDS)	2.0 ± 1.0	1.3 ± 0.6	≤ 10^−6^	F > M
Ethnicity (%)^c^:				
Asian/African American/Caucasian/Other	269/631/2650/346	282/629/2975/363	NS	F = M
Body Mass Index (BM1) *z*-scores^d^	0.23 ± 5.6	0.15 ± 14.1	NS	F = M

a Measured by *χ*^2^-test or *t*-test: NS “=” not significantly different by *p* = 0.05; ‘ *<* ‘ or ‘ *>* ‘ significantly different at *p* ≤ 0.05.

b Head size was measured after being affinely registered to the SRI24 template.

c Individuals who self-identified as Hispanic were included in the Caucasian group: 493 girls and 574 boys.

d *z*-Scores of the BMI (instead of percentile) are calculated by the *pygrowup* toolbox (pygrowup, 2017) for each individual to enable group comparison using *t*-test.

**Table 2:** Scores of cognitive tests (mean ± standard deviation).

Test	Cognitive process	Female (F) *N*=3895	Male (M) *N* = 4249	Cohen’s *d*	*p*-value*	Group difference*
NIH Toolbox: Flanker^®^	Cognitive control; attention	96.29 ± 13.37	97.09 ± 14.39	0.058	NS	F = M
NIH Toolbox: List Sorting Working Memory Test^®^	Working memory: categorization; information processing	101.68 ± 14.08	102.64 ± 14.68	0.067	0.038	F < M
NIH Toolbox Dimensional Change Card Sort^®^	Flexible thinking; concept formation; set shifting	98.89 ± 15.07	97.44 ± 15.54	0.095	0.3429e-2	F > M
NIH Toolbox Oral Reading Recognition Test^®^	Reading ability; language; academic achievement	104.45 ± 19.53	103.65 ± 18.52	0.042	NS	F = M
NIH Toolbox: Pattern Comparison Processing Speed^®^	Processing speed; information processing	96.70 ± 20.92	93.72 ± 22.18	0.140	0.1875e-4	F > M
NIH Toolbox: Picture Sequence Memory Test^®^	Visuospatial sequencing and memory	103.47 ± 16.47	100.62 ± 15.81	0.180	< 10^−6^	F > M
NIH Toolbox: Picture Vocabulary Test^®^	Language; verbal intellect	108.53 ± 16.98	109.35 ± 17.05	0.056	NS	F = M

* Measured by *χ*^2^-test or *t*-test: NS “=” not significant; ‘ *<* ‘ or ‘ *>* ‘ significant at *p* ≤ 0.05.

**Table 3:** Accuracy (Acc), true positive rate (TPR), true negative rate (TNR), area under the ROC curve (AUC) of different methods for predicting sex from MRIs.

Method	Acc	TPR	TNR	AUC
Ours (end-to-end deep learning)	89.6%	87.4%	91.5%	0.96
116 SRI24 volume scores				
Logistic Regression	74.2%	74.3%	74.0%	0.80
Support Vector Machine	74.2%	73.0%	75.5%	0.81
Random Forest	70.9%	66.7%	74.5%	0.75
906 Destrieux Parcellation Measures				
Logistic Regression	80.0%	80.8%	79.2%	0.88
Support Vector Machine	79.1%	78.1%	79.9%	0.84
Random Forest	74.2%	72.2%	76.0%	0.79

**Table 4:** *p*-Values of the correlation and mediation analysis with respect to the NIH Toolbox Scores. Correlation analysis was examined by Pearson’s *R* Mediation analysis examined the indirect effect of NIH Toolbox scores on sex prediction; Significant mediation effect (*p <* 0.05 for all 3 conditions of the partial mediation model) is marked by bold typeface. NS denotes *not significant*

Test	Correlation	Mediation		
	Prediction score	Observed sex (Condition 1)	Prediction score (Condition 2)	Correlation reduction (Condition 3)
NIH Toolbox Flanker^®^	NS	NS	NS	NS
NIH Toolbox List Sorting Working Memory Test^®^	0.001	**0.03817**	**0.0037**	**0.0005**
NIH Toolbox Dimensional Change Card Sort^®^	NS	0.00342	0.011	NS
NIH Toolbox Oral Reading Recognition Test^®^	NS	NS	0.036	NS
NIH Toolbox Pattern Comparison Processing Speed^®^	0.0025	0.00002	NS	NS
NIH Toolbox Picture Sequence Memory Test^®^	0.00001	< 10^−6^	NS	< NS
NIH Toolbox Picture Vocabulary Test^®^	0.0309	NS	NS	0.018

**Fig. 7.**
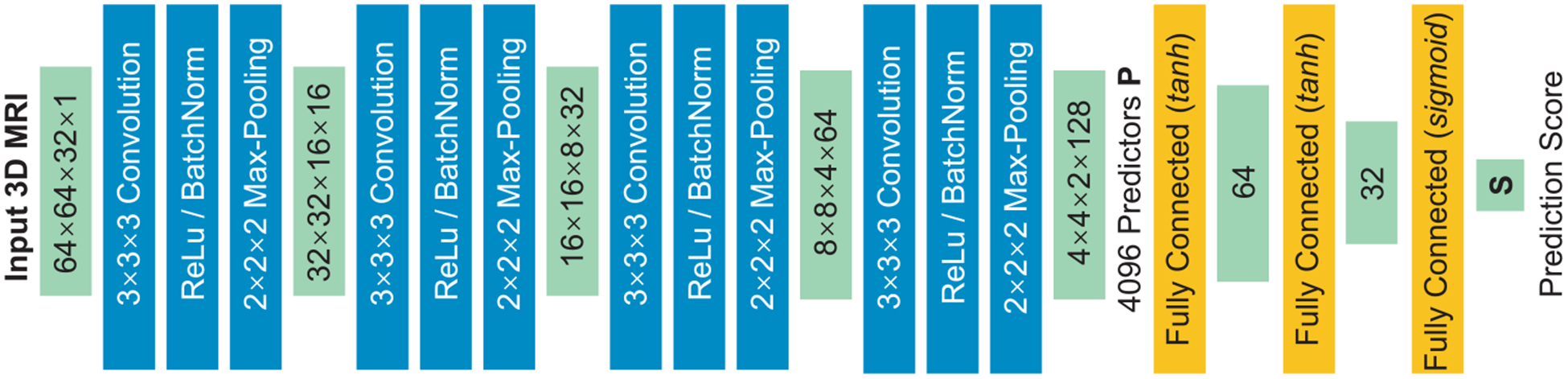
Architecture of our deep learning model.

**Fig. C.1.**
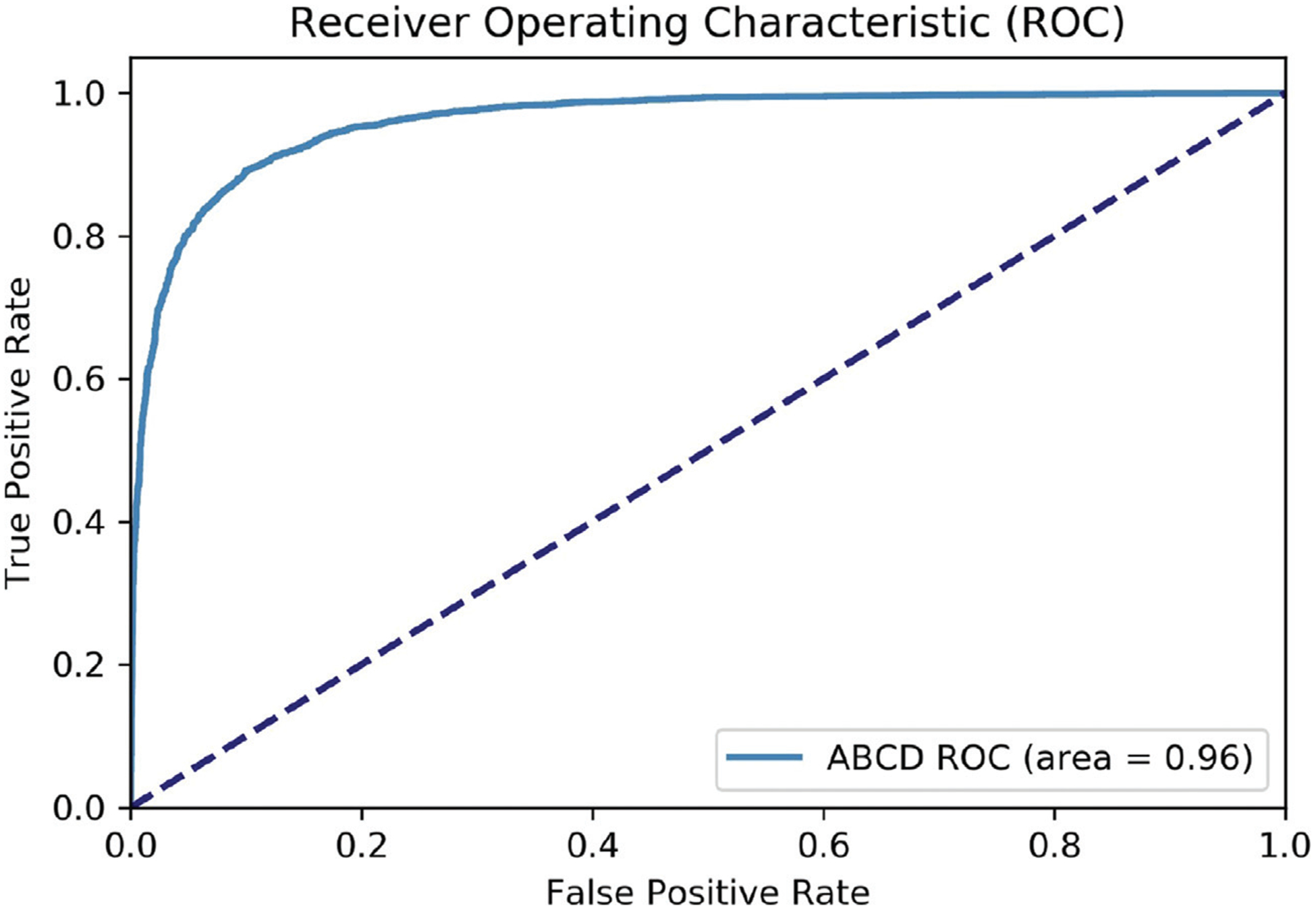
Receiver Operating Characteristics (ROC) curve of the classifier differentiating boys and girls based on MR images. The blue curve shows the results of the model based on ABCD data.

## Appendix A. Descriptions of the NIH Toolbox^®^ Cognitive Tests

The NIH Toolbox^®^ cognition measures were developed as part of the NIH Blueprint for Neuroscience Research (http://www.nihtoolbox.org). The tests assess episodic memory, executive function, attention, working memory, processing speed, and language abilities, enabling generation of composite scores (Gershon et al., 2013b; Hodes et al., 2013). Use of a common tool for cognitive assessment valid for ages spanning the ABCD cohort’s current and future range allows for longitudinal tracking of the developmental trajectories of this cohort in addition to harmonization and comparison of cognitive performance with numerous other studies. The tasks were selected based on a consensus building process and developed and validated using assessment methods that included item response theory (IRT) and computerized adaptive testing (CAT) where appropriate and feasible. Each Toolbox^®^ task produces a number of scores, some of which are adjusted for age, sex, and ethnicity. All tasks provide raw scores, uncorrected standard scores, and age-corrected standard scores based on a normative sample of 2917 children and adolescents (Casaletto et al., 2015). This study used age-corrected measures to compare the two cohorts of boys and girls, as there was a significant difference between our two cohorts. These tests are comprehensively described elsewhere (Luciana et al., 2018) and briefly below.

1. *Language/Vocabulary Comprehension:* The Toolbox Picture Vocabulary Task^®^ (TPVT) is a variant of the Peabody Picture Vocabulary Test (PPTV) (Gershon et al., 2014; 2013a; Mungas et al., 2014).
2. *Language/Reading Decoding:* The Toolbox Oral Reading Recognition Task^®^ (TORRT) asks individuals to pronounce single letters or words presented in the middle of the iPad screen (Gershon et al., 2014; 2013a) and measures exposure to language materials and cognitive skills involved in reading.
3. *Processing Speed:* The Toolbox Pattern Comparison Processing Speed Test^®^ (TPCPST) (Carlozzi et al., 2015; 2014; 2013) was modeled on the Pattern Comparison Task developed by Salthouse (Salthouse et al., 1991) and is a measure of rapid visual processing.
4. *Working Memory:* The Toolbox List Sorting Working Memory Test^®^ (TLSWMT) is a variant of the letter-number sequencing test (Gold et al., 1997) that uses pictures rather than words or letters (Tulsky et al., 2013, 2014).
5. *Episodic Memory:* The Toolbox Picture Sequence Memory Test^®^ (TPSMT) was modeled after memory tests asking children to imitate a sequence of actions with props developed by Bauer et al. (2013) and Dikmen et al. (2014b)
6. *Executive Function/Attention/Inhibition:* The Toolbox Flanker Task^®^ (TFT), a variant of the Eriksen Flanker task (Eriksen and Eriksen, 1974), was adapted from the Attention Network Task (Fan et al., 2002; Rueda et al., 2004) and assessed response inhibition.
7. *Executive Function/Cognitive Flexibility:* The Toolbox Dimensional Change Card Sort Task^®^(TDCCS) was based on the work of Zelazo and colleagues (Zelazo, 2006) and measures problem solving and cognitive flexibility.

## Appendix B. Deep learning model architecture and hyperparameters

Input to the deep learning model was the 3D MRI of one hemisphere of size 64 × 64 × 32. The predictor extraction network contained 4 stacks of 3 × 3 × 3 convolutional layers, ReLu activation, batch normalization, and 2 × 2 × 2 max-pooling layers. The size of feature channel for the 4 convolution layers was (16,32,64,128). Then the resulting 4096 features were fed into a set of fully connected layers (Multi-Layer Perceptron) classifier composed of three Fully Connected (FC) layers of dimension (64,32,1). tanh activation was used for the first two FC layers, and sigmoid activation was used for the last FC layer resulting in the final prediction score **S** ∈ [0, 1]. An L2 regularization of weight 0.1 was applied to the FC layers (see Fig. 7).

## Appendix C. Receiver operating characteristic curve

As included in the main paper, our deep learning framework led to an accuracy of nearly 90% for predicting the sex of individuals based their structural MRI data. The receiver operating characteristic (ROC) curve of this classification model is depicted in Appendix Fig. C.1, which shows an area under the curve (AUC) of 0.96.
